# Effect of Cooking Techniques on the *in vitro* Protein Digestibility, Fatty Acid Profile, and Oxidative Status of Mealworms (*Tenebrio molitor*)

**DOI:** 10.3389/fvets.2021.675572

**Published:** 2021-06-04

**Authors:** Simone Mancini, Simona Mattioli, Simone Paolucci, Filippo Fratini, Alessandro Dal Bosco, Tiziano Tuccinardi, Gisella Paci

**Affiliations:** ^1^Department of Veterinary Sciences, University of Pisa, Pisa, Italy; ^2^Interdepartmental Research Center “Nutraceuticals and Food for Health”, University of Pisa, Pisa, Italy; ^3^Department of Agricultural, Food and Environmental Science, University of Perugia, Perugia, Italy; ^4^Department of Pharmacy, University of Pisa, Pisa, Italy

**Keywords:** protein digestibility, frying, microwave, tocols, carbonyl, TBARS, oven cooking

## Abstract

*Tenebrio molitor (T. molitor)* (mealworm) larvae are one of the most promising insects for feed–food purposes. Mealworms are rich in several macro and micro nutritional elements and can be practically reared on side stream substrates. In this study, the effects of seven different cooking techniques were tested on the nutritional value of mealworms focusing the attention on protein digestibility, fatty acid (FA) profile, and oxidative status. Uncooked larvae (UC) were used as control and compared to two combinations of temperature/time in oven cooking (70°C for 30 min, OC70-30, 150°C for 10 min, OC150-10), two methods of frying (mealworms fried in sunflower oil as deep fry, DF, or pan fry, PF), microwaving (MW), boiling (in plastic bag under vacuum, BO), and steaming (ST). Proximate composition, *in vitro* digestibility (gastric and duodenal), FA profile, and oxidative status (tocopherol and tocotrienol, carbonyl, and lipid oxidation) were then tested. Cooking technique affected all the tested parameters. As expected, cooking affected proximate composition in relation to the method applied (dry matter increased after oven cooking and frying; lipids increased by frying). *In vitro* digestion revealed the highest value for the OC70-30 method, followed by UC and ST. Deep frying revealed the worst digestibility percentage. FA profile was deeply affected by the cooking technique, with general decrease in SFA and MUFA. The highest modifications in FA profile were revealed in ST larvae with an increased percentage of linoleic acid linked to the lowering of SFA and MUFA contents. Furthermore, deep frying larvae in sunflower oil increased the relative abundance of PUFAs. Tocols values were higher in DF and MW groups than PF (about 6-fold more) and all other groups (7-fold more). Carbonyls increased with oven cooking (OC150-10 and OC70-30), whereas the values were lower with frying and similar to ST and UC. Lipid oxidation was highest as well in OC150-10 but similar to frying methods (DF and PF). Based on the obtained results, it can be concluded that mealworm larvae surely meet human nutritional requirements, but the cooking method must be carefully chosen to maintain a high nutritional value.

## Introduction

In the last few years, attention on edible insects has grown in relation to the increasing request of nutrient foods (1). Alternatives to the ongoing animal productions are required to sustain the increase of human population while lowering environmental impact. Insects could meet human nutrient requirements and increase the production yields without negatively impacting the environment. One of the main intrinsic potential of insect production lies on their capacity to be reared on unemployed feeds, also called substrate, that nowadays are not employed in conventional animal productions or in some cases are even treated as waste materials (2, 3). Indeed, insects could be fed on low-value feed contributing to decrease the environmental negative impact of animal farming and increasing the circular economy, with positive benefits on the entire food chain production (4, 5). Several research studies highlighted the potential of insects to bio convert low-value substrates into high-value nutrient products, with different outcomes in relation to the dualism insect substrate. Despite the increased attention shown by the scientific world, consumers' acceptance of edible insects in Europe is still low (6). Several researches highlighted that acceptability and consumers' awareness could be increased by providing information about insects' farming features, their sustainability and environmental perspectives, along with introducing insects in daily products (7–9). Processing of insects is a crucial step to make them utilizable as food and reach consumers' tables. As one of the last steps before consumption, cooking plays a key role in the nutritional value of food. Safe and healthy cooking methods may contribute to increase availability of edible insects in daily food consumption (10, 11).

Cooking may improve the sensory appeal of products, modifying color, texture, and flavors while affecting bioavailability of certain nutrients in the digestive tract. Heat treatment may also induce oxidations, proteolysis, lipolysis, and losses of susceptible molecules (11, 12).

Among edible insects, mealworm (*Tenebrio molitor* L. 1758; Coleoptera Tenebrionidae) is one of the most studied and produced insects for human consumption in Europe due to its features in rearing management, environmental sustainability, and nutritional value. Mealworm is the first edible insect that has achieved an important milestone for the EU insect sector as it was positively assessed by the European Food Safety Authority (EFSA) as novel food following the Regulation (EU) 2015/2283. Indeed, recently, EFSA released the first scientific opinion about insects as novel food (13) in which the EFSA Panel affirmed that dried mealworm (*T. molitor* larva) could be considered as a safe novel food. Considering this scientific opinion, a considerable expectation arises on mealworms and their potential will be released in the next years following market developments. From a nutritional point of view, mealworm larvae are quite balanced in macronutrients with high level of proteins (about 50% on dry matter basis) and lipids (about 33% on dry matter basis) and a good composition of essential amino acids, vitamins, and minerals (4, 14–17). Rearing substrate could affect development of mealworms (18, 19) as well as partially affect protein concentration and fatty acid (FA) composition (also ratio n6/n3) (4, 20, 21). Similarly, processing (i.e., cooking methods) of the larvae could affect the chemical composition of the products, both nutritional and quality characteristics (11, 22, 23).

In this study, the effects of seven different cooking techniques (two combinations temperature/time in oven cooking, two types of frying methods, microwave, boiling, and steaming) were tested on the nutritional values of mealworms focusing the attention on the *in vitro* protein digestibility, FA profile, and oxidative status.

## Materials and Methods

### Insect Rearing

*Tenebrio molitor* larvae were fed with brewery spent grains and bread as former foodstuff. Brewery spent grains were directly collected from a local brewery and immediately frozen at −20°C, and bread was collected from a local market shop at the closure of the shop as daily remains. Both breweries spent grains and bread were dried in an oven at 90°C (spent grains were previously thawed at 4°C for 18 h) until the excessive humidity was removed reaching respectively about 95 and 97% in dry matter. Then, spent grains and bread were finely ground and mixed in 1:1 ratio. Larvae were reared in plastic containers (39 × 28 × 14 cm) at the Department of Veterinary Sciences (University of Pisa, Italy) under a laboratory-scale production. Rearing environment was maintained at 25 ± 1°C with 50–60% of relative humidity. Adult beetles (1–2 weeks old) were placed for 1 week to deposit eggs and then were removed to leave larvae to hatch in the substrate. Feeds were added weekly if needed (*ad libitum*, weighted before adding) and potato slides were placed once a week to provide moisture. During the rearing period, three times per week, rearing boxes were visually monitored and eventually dead larvae were removed. Mealworms were then harvested when the first pupa was observed into the box and used as samples for experimental units. Larvae were starved for 48 h in in sterile plastic containers with plastic web on the base in order to separate feces (frass) and avoid fecal contact. Larvae were then killed by freezing at −20°C.

### Cooking Parameters

Larvae were cooked with seven different cooking methods as reported below. Two different combinations of temperature/time were selected for the oven cooking method, as well two types of frying were chosen.

- Oven cooking: oven was pre-heated at 70°C or 150°C and larvae were cooked in aluminum trays for 30 min (OC70-30) or 10 min (OC150-10).
- Frying: mealworms were fried in sunflower oil: either deep fry (300 ml of oil per 100 g of larvae, DF) or pan fry (30 ml of oil per 100 g of larvae, PF) for 2 min and dried on a paper towel.
- Microwave: larvae were cooked in a glass bowl at 800 W per 150 s (MW).
- Boiled: larvae were kept in a plastic bag under vacuum and inserted in boiling water for 30 min (BO).
- Steamed: larvae were cooked in a steamer for 10 min (ST).

Cooking loss was recorded and calculated as percentage of the decrease of weight before and after cooking. Raw larvae were used as control (uncooked, UC).

All the cooking sessions were performed in triplicate on three different experimental units.

### Proximate Composition

Dry matter content was determined by dehydration in a drying oven at 105°C until constant weight. Soxhlet extraction method was used to quantify lipid content using petroleum ether as solvent. Ash content was determined by incineration in a muffle furnace at 550°C. Crude protein content was determined by the Kjeldahl method. For protein-to-nitrogen conversion, two factors were used, 6.25 as normally calculated for meat an unknown samples and 4.76 as suggested by Janssen et al. (24) for insects. Proximate composition analyses were performed in triplicate for each sample.

### *In vitro* Protein Digestibility

**Figure 1:**
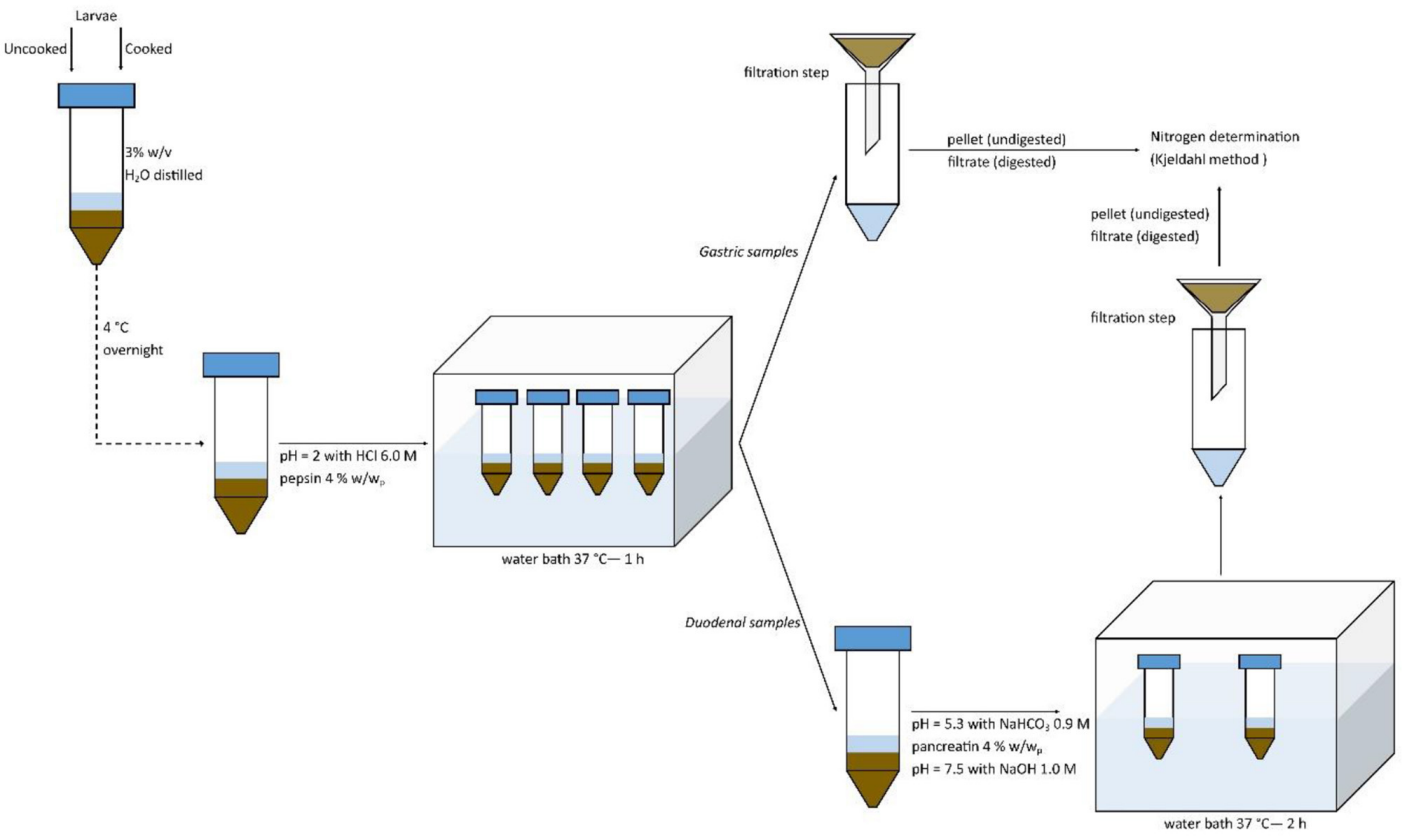
*In vitro* protein digestibility method.

*In vitro* protein digestibility was performed following the method of Lacroix et al. (25) with slight modifications, as reported in Figure 1. In detail, ground uncooked or cooked larvae were diluted in distilled water at a concentration of 30 g/L (3% w/v) and solubilized at 4°C overnight. Then, samples were adjusted to pH 2 with HCl 6 M pre-incubated at 37°C. Pepsin was added at the concentration of 4% enzyme: substrate, based on the ratio w/w of protein (determined by the Kjeldahl method right after cooking). Solutions were then incubated in a water bath at 37°C for 1 h in order to simulate gastric digestion. Samples were then split into two ways: the first ones were filtrated (filter paper Whatman 1) in order to obtain the pellet (undigested) and filtrate (digested) fractions after the *in vitro* gastric digestion; the second ones were adjusted to pH 5.3 with NaHCO_3_ 0.9 M, and then pancreatin was added at the concentration of 4% (w/w of protein basis). Solutions were then adjusted with NaOH 1.0 M at pH 7.5 and incubated at 37°C for 2 h in a water bath in order to simulate an *in vitro* duodenal digestion. Samples were then filtered to obtain digested (filtrate) and undigested (pellet) fractions. All the fractions were subjected to Kjeldahl determination. Digestibility values were calculated as percentage of the nitrogen contents of the fractions on the total sample's nitrogen content. Blanks containing only enzymes were simultaneously run and subtracted to the sample's determinations.

*In vitro* digestibility was performed in triplicate on each cooking technique and on uncooked larvae.

### FA Profile

The lipid extraction from larvae was performed in duplicate on each sample according to Folch et al. (26) and the esterification of FA was performed according to Christie (27). The trans-methylation procedure was conducted using eicosenoic acid methyl esters (Sigma-Aldrich, Germany) as internal standard. The recovery rate of the internal standard was 85% ± 5% in the larvae.

The FA composition was determined using a Varian gas chromatograph (CP-3800) equipped with a flame ionization detector and a capillary column of 100 m length × 0.25 mm × 0.2 μm film (Supelco, Bellefonte, PA, USA). Helium was used as the carrier gas with a flow rate of 0.6 ml/min. The split ratio was 1:20. The oven temperature was programmed as reported by Mattioli et al. (28). Individual fatty acid methyl ester (FAME) was identified by comparing the relative retention times of peaks in the sample with those of a standard mixture (FAME Mix Supelco, Sigma-Aldrich, Germany). The FA were expressed as % of total FA. The average amount of each FA was used to calculate the sum of the total saturated (SFA), total monounsaturated (MUFA), and total polyunsaturated (PUFA) FA form n-3 and n-6 series. Even n6/n3 FA ratio was calculated.

In the insect samples, some nutritional indexes of lipids were evaluated as reported below.

The index of peroxidability (IP) was calculated according to Arakawa and Sagai (29):

$$\begin{array}{l} \text{IP}=\left(\text{\%monoenoic}\times 0.025\right)+\left(\text{\%dienoic}\times 1\right)+\left(\text{\%trienoic}\times 2\right)\\ \;+\left(\text{\%tetraenoic}\times 4\right)+\left(\text{\%pentaenoic}\times 6\right)+\left(\text{\%hexaenoic}\times \;8\right). \end{array}$$

Indexes of atherogenicity (IA) and thrombogenicity (IT) were calculated according to Ulbricht and Southgate (30). In particular:

$$\begin{array}{l} \text{IA}=\left(\text{C12}:\text{0}+\text{C14}:\text{0}\times \text{4}+\text{C16}:\text{0}\right)\text{/}[\left(\text{MUFA}+\Sigma \text{n6}\right)\\ \;\text{+}\Sigma \text{n3})];\\ \text{IT}=\left(\text{C14}:\text{0 + C16}:\text{0 + C18}:\text{0}\right)\text{/}[(\text{MUFA}\times \text{0}.\text{5}+\Sigma \text{n6}\times \text{0}.\text{5}\\ \;+\Sigma \text{n3}\times \text{3})+\Sigma \text{n3/}\;\Sigma \text{n6})]. \end{array}$$

### Oxidative Status

The tocopherol (δ-, γ-, and α-isoforms) and tocotrienol (γ- and α-isoforms) contents of the samples were quantified by a high-performance liquid chromatography (HPLC) system, according to Zaspel and Csallany (31). About 3 g of larvae was saponified in 60 g/100 ml KOH in ethanol in a thermostat bath at 70°C for 30 min. Then, the content was sonicated and extracted twice with 15 ml of n-hexane/ethyl acetate (9:1, v/v). After collecting the upper phase, the samples were nitrogen dried and then reconstituted in 200 μl of acetonitrile. Fifty microliters was injected into the HPLC system (Perkin Elmer series 200, equipped with an autosampler system, model AS 950–10, Tokyo, Japan) on a Synergy Hydro-RP column (4 μm, 4.6 × 100 mm; Phenomenex, Bologna, Italy). A Fluorometric Detector (FD) (model, FP-1525; Jasco, Tokyo, Japan; excitation and emission wavelengths of 295 and 328 nm, respectively) was used to identify the different isoforms. External calibration curves were used to quantify isoforms by increasing amounts of pure tocopherols in ethanol. The sum of tocopherols and tocotrienols were named tocols.

Carbonyl derivatives of proteins were detected according to Mattioli et al. (32). Briefly, the pellets from trichloroacetic acid (TCA) extracts were mixed with 1 ml of 10 mM DNPH in 2 M HCl. Samples were incubated for 1 h at RT and then centrifuged at 13,000 × *g* for 5 min. Supernatants were discarded and pellets were washed three times with 1 ml of ethanol–butylacetate (1:1, v/v) to remove unreacted DNPH. Pellets were then dissolved in 1.5 ml of 6 M guanidine-HCl and centrifuged as above to pellet insoluble particles. The carbonyl content of the resulting supernatants was evaluated spectrophotometrically at 370 nm using a molar extinction coefficient of 22,000 1/M^*^cm; values were expressed as nanomoles of carbonyl per milligram of protein in the guanidine chloride solution. Protein concentrations were measured *via* the Bradford method with Coomassie Brilliant Blue G-250 (33), using bovine serum albumin as standard.

Lipid oxidation was evaluated using a spectrophotometer set at 532 nm (Shimadzu Corporation UV-2550, Kyoto, Japan) that measured the absorbance of TBARS and a 1,1,3,3-tetraethoxypropane calibration curve (34). Oxidation products were quantified as malondialdehyde equivalents (μg MDA/g).

### Statistical Analysis

Proximate composition, cooking loss, *in vitro* protein digestibility, FA profile, and oxidative status were statistically analyzed using a one-way ANOVA with regard to the cooking technique factor (UC, OC70-30, OC150-10, DF, PF, MW, BO, and ST). Statistical significance was set at 0.05 and 0.001 and differences were assessed using Tukey's test. R free statistical software was used (35).

## Results

### Proximate Compositions and Cooking Losses

**Figure 2:**
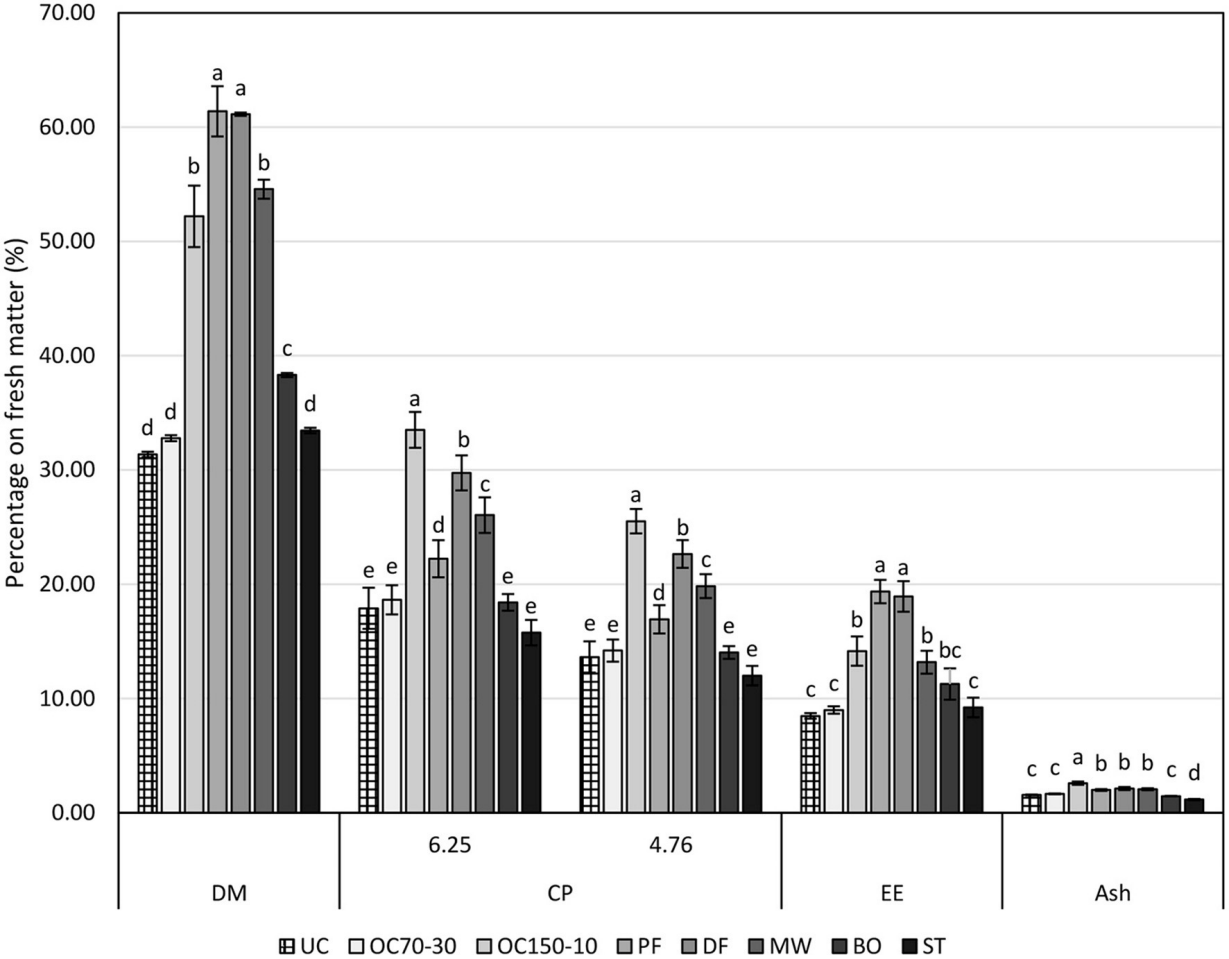
Proximate compositions (on fresh matter, %) of uncooked larvae and larvae cooked with different techniques. DM, dry matter; CP, crude protein (protein-to-nitrogen conversion factors of 6.25 and 4.76); EE, ether extract; UC, uncooked; OC70-30 oven cooked at 70°C for 30 min; OC150-10, oven cooked at 150°C for 10 min; PF, pan fried (30 ml oil per 100 g of larvae, 2 min); DF, deep fried (300 ml of oil per 100 g of larvae, 2 min); MW, cooked in microwave (800 W per 150 s); BO, boiled (plastic bag under vacuum for 30 min); ST, steamed for 10 min. Different letters show statistically significant differences among samples for each determination (DM, CP_6.25, CP_4.76, EE and Ash) at *P* < 0.05.

Cooking techniques affected proximate compositions of larvae (Figure 2). Dry matters of fried larvae (PF and DF) were higher than the other samples, followed by oven cooked at 150°C (OC150-10) and microwaved (MW), boiled ones (BO) and by oven cooked at 70°C (OC70-30), steamed (ST), and uncooked (UC). These losses of humidity were also detected by cooking losses that were, respectively, detected as 5.05, 35.91, 16.38, 25.27, 27.22, and 3.35% for OC70-30, OC150-10, PF, DF, MW, and BO. Steamed larvae (ST) reported cooking loss as a negative value, −15.39%, as during the cooking step, larvae gained weight instead of losing weight. Cooking losses indeed affected all the proximate compositions as the higher cooking technique in terms of cooking loss, i.e., OC150-10, DF, and MW, reported higher concentration of crude proteins followed by PF. No significant variation to the UC larvae was highlighted for the other cooking techniques (OC70-30, BO, and ST). As expected, frying increased the percentage of lipids in the final products, as revealed by DF and PF ether extract values. As reported for crude protein contents, cooking losses affected the lipid percentages too. Oven cooking at 150°C (OC150-10) and MW larvae reported higher contents in ether extract than UC, OC70-30, and ST. Boiling larvae slightly increased the ether extract concentration in relation to the raw larvae. Ash concentration was mainly affected by the cooking technique, highlighting a higher concentration in OC150-10 followed by PF, DF, and MW, and then by UC, OC70-30, and BO larvae. Steamed larvae (ST) due to humidity increase revealed lower ash content than UC ones.

Proximate composition of the larvae was also reported as percentage on dry matter (DM, Table 1). Crude protein content revealed, as percentage on DM, to be higher in oven-cooked larvae at 150°C, as consequence of humidity lost. Raw larvae (UC) and OC70-30 crude protein contents were similarly high and comparable to DF, MW, BO, and ST. The lowest value was shown by PF larvae. Frying indeed increased ether extract as DM percentage; as a consequence, PF and DF showed around one-third of DM as lipids. No statistical differences were highlighted between the other samples. Ash content showed a similar trend to that of crude protein and seems to be mainly affected by cooking losses.

**Table 1:** Proximate compositions (on dry matter, %) of uncooked larvae and larvae cooked with different techniques.

	**UC**	**OC70-30**	**OC150-10**	**PF**	**DF**	**MW**	**BO**	**ST**	**RMSE**	***P***
CP_6.25	57.07^ab^	56.85^ab^	64.20^a^	36.21^c^	48.65^b^	47.73^b^	48.07^b^	47.12^b^	7.305	<0.001
CP_4.76	43.47^ab^	43.30^ab^	48.89^a^	27.58^c^	37.05^b^	36.35^b^	36.61^b^	35.89^b^	5.564	<0.001
EE	27.04^b^	27.44^b^	27.11^b^	31.54^a^	30.97^a^	24.14^b^	29.41^b^	27.56^b^	5.384	0.049
Ash	4.95^a^	5.05^a^	4.97^a^	3.25^c^	3.48^bc^	3.78^b^	3.81^b^	3.46^bc^	0.173	<0.001

*DM, dry matter; CP, crude protein (protein-to-nitrogen conversion factors of 6.25 and 4.76); EE, ether extract*.

*UC, uncooked; OC70-30 oven cooked at 70°C for 30 min; OC150-10, oven cooked at 150°C for 10 min; PF, pan fried (30 ml oil per 100 g of larvae, 2 min); DF, deep fried (300 ml of oil per 100 g of larvae, 2 min); MW, cooked in microwave (800 W per 150 s); BO, boiled (plastic bag under vacuum for 30 min); ST, steamed for 10 min*.

*Different letters in the same row show statistically significant differences among samples at P <0.05*.

### *In vitro* Protein Digestibility

**Figure 3:**
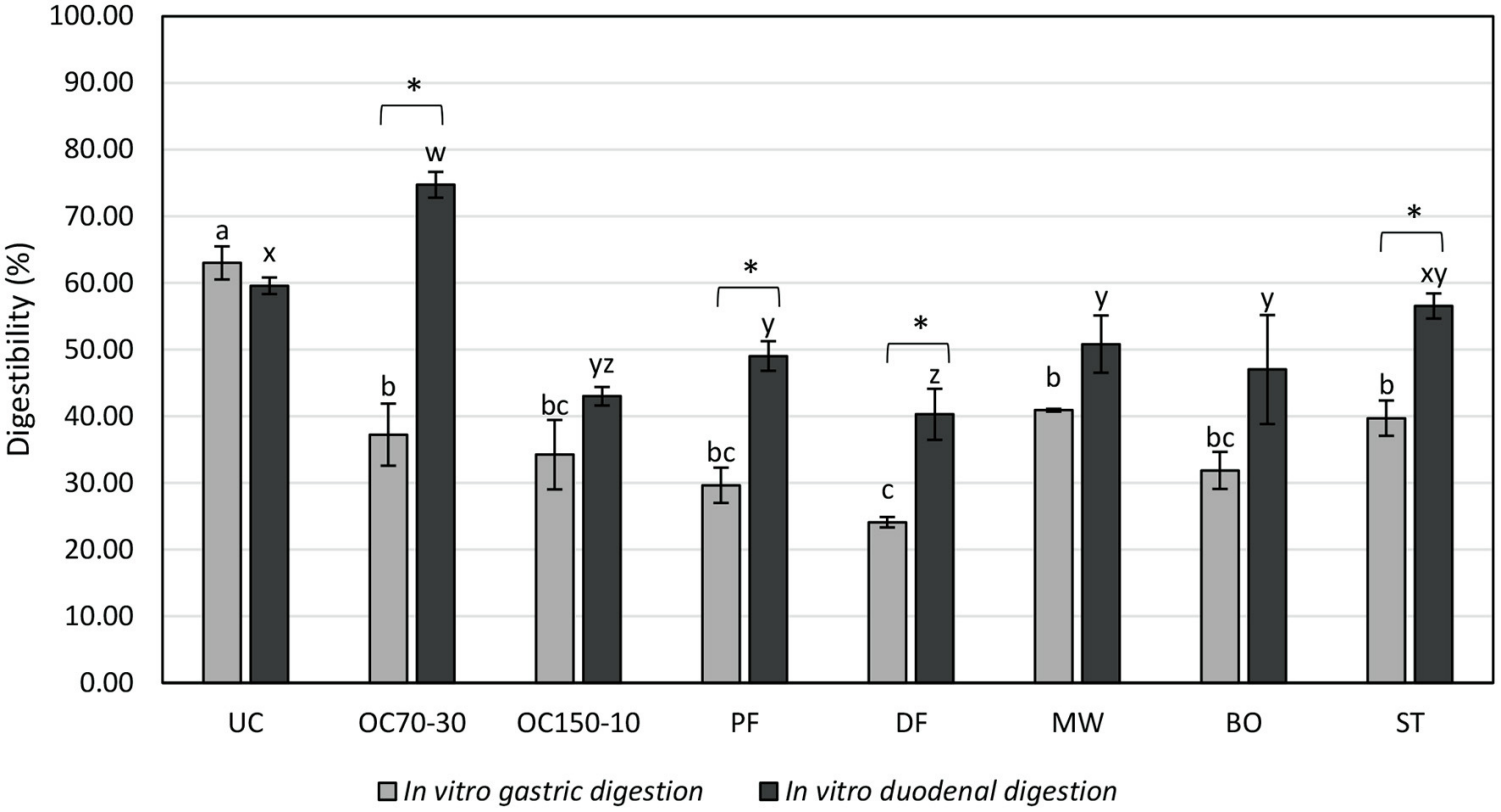
*In vitro* digestibility (filtrate nitrogen content on the total sample nitrogen content, %) of uncooked larvae and larvae cooked with different techniques. UC, uncooked; OC70-30 oven cooked at 70°C for 30 min; OC150-10, oven cooked at 150°C for 10 min; PF, pan fried (30 ml oil per 100 g of larvae, 2 min); DF, deep fried (300 ml of oil per 100 g of larvae, 2 min); MW, cooked in microwave (800 W per 150 s); BO, boiled (plastic bag under vacuum for 30 min); ST, steamed for 10 min. ^a,b,c^Letters show statistically significant differences among samples for *in vitro* gastric digestion at *P* < 0.05. ^w,x,y,z^Letters show statistically significant differences among samples for *in vitro* duodenal digestion at *P* < 0.05. *Shows statistically significant differences between *in vitro* gastric and duodenal digestions among each treatment at *P* < 0.05.

*In vitro* digestibility values, reported as percentage of nitrogen content of the filtrates on the total samples, are reported in Figure 3.

After *in vitro* gastric digestion, uncooked samples revealed the highest digestibility value, followed by OC70-30, MW, and ST samples. Deep fried larvae (DF) showed the lowest digestibility after *in vitro* gastric digestion. After *in vitro* duodenal digestion, the highest value was determined in OC70-30 larvae, followed by UC, ST, and then PF, MW, BO, and OC150-10 samples. As reported before for *in vitro* gastric digestion, DF larvae revealed the worst digestibility percentage. Statistical differences in *in vitro* digestibility between gastric and duodenal samples were not shown by UC, OC150-10, MW, and BO samples even if for all the cooking techniques a numerical increase in digestibility was revealed (not for UC). A statistically significant increase in digestibility of OC70-30, PF, DF, and ST samples was highlighted with values of +37.48%, +19.39%, +16.18%, and +16.83%, respectively. Interestingly, OC70-30 digestibility doubled after *in vitro* duodenal digestion in relation to gastric ones.

### FA Profile

Many differences were found on FA proportion of cooked larvae (Table 2). All cooking processes decreased the content of SFA (mainly constituted by palmitic acid, C16:0) with respect to the uncooked larvae. The MUFA showed less variations with similar value in UC, OC150-10, PF, MW, and BO (*P* > 0.05); ST showed the lowest value (32.24% of total FA). The main differences were due to the palmitoleic (C16:1) and oleic (C18:1) acid proportion, which were the most representative FA in OC70-30 and UC, respectively (C16:1 was 7.91% in OC70-30 and C18:1 was 40.68% in UC).

**Table 2:** Fatty acid profile (%) of uncooked larvae and larvae cooked with different techniques.

	**UC**	**OC70-30**	**OC150-10**	**PF**	**DF**	**MW**	**BO**	**ST**	**RMSE**	***P***
C12:0	0.24	0.19	0.19	0.35	0.24	0.36	0.24	0.24	0.063	0.470
C14:0	3.53^A^	2.12^B^	3.23^A^	3.53^A^	0.05^C^	0.12^C^	3.07^A^	0.09^C^	0.163	<0.001
C16:0	21.42^A^	19.58^A^	19.16^B^	19.05^B^	9.39^C^	19.27^B^	18.81^B^	6.96^D^	0.594	<0.001
C18:0	4.54^a^	3.14^b^	3.54^b^	3.53^b^	3.62^ab^	3.82^ab^	3.64^b^	3.50^b^	0.128	0.002
C20:0	0.65^B^	2.33^A^	0.23^B^	0.06^B^	0.47^B^	0.04^B^	0.14^B^	0.03^B^	0.180	<0.001
SFA	30.37^A^	27.35^B^	26.35^B^	26.52^B^	13.76^D^	23.61^C^	25.90^BC^	10.82^D^	0.472	<0.001
C14:1cis9	0.33^C^	1.04^A^	0.11^DE^	0.15^D^	0.77^B^	0.14^D^	0.10^DE^	0.02^E^	0.019	<0.001
C16:1cis9	1.68^B^	7.91^A^	1.63^B^	1.84^B^	0.60^C^	1.73^B^	1.74^B^	0.19^C^	0.137	<0.001
C18:1cis9 n9	40.68^A^	25.11^E^	39.81^AB^	39.24^AB^	34.82^C^	37.99^B^	38.54^AB^	32.00^D^	0.436	<0.001
C20:1cis+trans11	0.15^B^	0.86^A^	0.15^BC^	0.15^B^	0.05^D^	0.12^C^	0.04^E^	0.03^E^	0.005	<0.001
MUFA	42.83^a^	34.92^c^	41.69^ab^	41.38^ab^	36.23^c^	39.98^b^	40.42^ab^	32.24^d^	0.449	<0.01
C18:2cis n6 LA	14.49^C^	11.94^C^	28.11^B^	27.12^B^	47.22^A^	14.20^C^	28.38^B^	51.43^C^	1.267	<0.001
C20:4n6 AA	0.58^B^	1.73^A^	0.03^D^	0.05^D^	0.18^C^	0.09^D^	0.05^D^	0.24^C^	0.012	<0.001
C22:5n6	0.20	0.06	0.01	0.05	0.04	0.02	0.00	0.01	0.065	0.437
n6	15.27^C^	13.73^C^	28.15^B^	27.22^C^	47.44^A^	14.31^C^	28.43^B^	51.68^A^	1.266	<0.001
C18:3 n3 ALA	0.71^C^	3.15^B^	1.37^B^	1.42^B^	0.55^C^	0.15^D^	1.42^B^	0.64^C^	0.058	<0.001
C20:5n3 EPA	1.12^B^	1.48^A^	0.03^C^	0.10^C^	0.03^C^	0.05^C^	0.06^C^	0.02^C^	0.036	<0.001
C22:5n3 DPA	0.11^b^	2.05^a^	0.03^d^	0.04^bc^	0.01^d^	0.04^d^	0.04^bc^	0.01^d^	0.014	0.091
C22:6n3 DHA	0.84^B^	2.31^A^	0.00^C^	0.05^C^	0.01^C^	0.01^C^	0.05^C^	0.03^C^	0.066	<0.001
n3	2.78^B^	9.00^A^	1.43^CD^	1.61^C^	0.60^DE^	0.23^E^	1.57^C^	0.69^DE^	0.153	<0.001
PUFA	18.05^D^	22.73^C^	29.58^BC^	28.83^BC^	48.04^A^	14.55^D^	30.00^B^	52.38^A^	1.268	<0.001
tot	91.25^AB^	85.00^BC^	97.62^A^	96.73^A^	98.03^A^	78.14^C^	96.33^A^	95.44^A^	1.469	<0.001
others	8.75^BC^	15.00^AB^	2.38^C^	3.27^C^	1.97^C^	21.86^A^	3.67^C^	4.56^C^	1.469	<0.001
n6/n3	5.49^DE^	1.53^E^	19.71^C^	16.92^CD^	79.41^A^	62.46^B^	18.10^C^	74.76^AB^	2.835	<0.001
IP	14.08^B^	39.67^A^	0.35^C^	1.20^C^	0.32^C^	0.53^C^	0.98^C^	0.37^C^	0.741	<0.001
IA	0.59^A^	0.49^B^	0.45^B^	0.48^B^	0.12^D^	0.37^C^	0.44^BC^	0.09^D^	0.014	<0.001
IT	0.78^A^	0.48^C^	0.66^B^	0.67^B^	0.30^D^	0.83^A^	0.65^B^	0.24^D^	0.016	<0.001

*UC, uncooked; OC70-30 oven cooked at 70°C for 30 min; OC150-10, oven cooked at 150°C for 10 min; PF, pan fried (30 ml oil per 100 g of larvae. 2 min); DF, deep fried (300 ml of oil per 100 g of larvae. 2 min); MW, cooked in microwave (800 W per 150 s); BO, boiled (plastic bag under vacuum for 30 min); ST, steamed for 10 min*.

*^a, b, c, d^Letters show statistically significant differences among samples at P <0.01*.

*^A, B, C, D, E^Letters show statistically significant differences among samples at P <0.001*.

The PUFA content followed the rank ST and DF > BO, OC150-10, and PF > OC70-30 > UC, and MW. The most representative FA were the LA for n6 series and ALA for n3 series. Among the long-chain FA (LCP), strong differences were recorded; however, the OC70-30 showed the highest values for both LCP n3 and n6.

The n6/n3 ratio was interestingly lower in OC70-30 (1.53) whereas the highest value was recorded in DF treatment (79.41), mainly due to the higher proportion of LA (2-fold higher with respect to the average of the other treatments). The IP reached the highest value in OC70-30, followed by UC; all the other cooking processes showed lower values. IA and IT were also widely reduced in cooked samples with respect to the fresh larvae.

### Oxidative Status

The tocols content were widely affected by moisture of cooked samples and then the values were reported on DM basis (Table 3). The α-tocopherol isoform was the most abundant in all samples. Tocols values were higher in DF and MW groups than in PF (about 6-fold more) and all other groups (7-fold more).

**Table 3:** Antioxidant content (μg/g DM) and oxidative status (Thiols, μmol SH-group/g DM; carbonyls, nmol/mg proteins calculated on DM basis; TBARS μg of MDA/g DM) and of uncooked larvae and larvae cooked with different techniques.

	**UC**	**OC70-30**	**OC150-10**	**PF**	**DF**	**MW**	**BO**	**ST**	**RMSE**	***P***
**Antioxidants**
γ-Tocotrienol	0.82^b^	1.11^a^	1.23^a^	0.55^b^	1.27^a^	0.77^b^	0.94^b^	1.14^a^	0.081	0.038
α-Tocotrienol	0.02	0.03	0.03	0.01	0.03	0.02	0.03	0.03	0.004	0.644
δ-Tocopherol	0.01	0.02	0.00	0.03	0.26	0.24	0.00	0.00	0.064	0.466
γ-Tocopherol	0.15^b^	0.10^b^	0.10^b^	0.17^b^	1.18^a^	1.04^a^	0.09^b^	0.13^b^	0.07	0.460
α-Tocopherol	1.55^C^	1.26^C^	1.20^C^	7.79^B^	17.12^A^	17.19^A^	1.86^C^	1.24^C^	3.740	<0.001
Tocols	2.56^C^	2.52^C^	2.56^C^	8.55^B^	17.40^A^	17.40^A^	2.93^C^	2.55^C^	47.490	<0.001
**Oxidative status**
Carbonyls	8.23^D^	95.38^A^	117.02^A^	7.95^D^	7.91^D^	55.99^B^	15.22^C^	8.87^D^	5.613	<0.001
TBARS	2.85^c^	3.97^b^	5.68^a^	4.02^b^	5.70^a^	2.99^c^	2.50^c^	2.38^d^	0.608	0.018

*UC, uncooked; OC70-30, oven cooked at 70°C for 30 min; OC150-10, oven cooked at 150°C for 10 min; PF, pan fried (30 ml oil per 100 g of larvae, 2 min); DF, deep fried (300 ml of oil per 100 g of larvae, 2 min); MW, cooked in microwave (800 W per 150 s); BO, boiled (plastic bag under vacuum for 30 min); ST, steamed for 10 min*.

*^a, b, c, d^Letters show statistically significant differences among samples at P < 0.05*.

*^A, B, C, D, E^Letters show statistically significant differences among samples at P < 0.001*.

Protein oxidative status was significantly different in all groups (*P* < 0.001), following the rank: OC50-10 and OC70-30 > MW > BO > others, whereas the lipid oxidation showed significantly higher values in OC150-10 and DF with respect to the other groups.

## Discussion

Cooking may improve the sensory characteristics of food and plays a key role in assuring safety parameters. Noteworthy, it may also affect nutritional value, both positively and negatively. Heat treatments might decrease the amount of thermolabile compounds (such as PUFAs or vitamins) and induce interactions between proteins themselves or with other oxidizing agents [sugars, polyphenols, tannins, or solvents; (36)] affecting the total digestibility. Moreover, high temperatures could denature protein unfolding polypeptide chains increasing susceptibility to enzymes and inactivate antinutritional compounds that may inhibit specific enzymes (36, 37). At the same time, excessive high temperatures can reduce digestibility of proteins inducing amino acid reactions that vane enzyme digestion (37).

### Proximate Composition

Mealworms show a high content of proteins (about 20% of fresh matter) and lipids (about 14% of fresh matter). These two parameters make mealworm nutritional value comparable to other conventional animals' products such as meat, eggs, or milk. The obtained ranges of nutrients, beside the cooking technique, are in agreement with other previously published researches on mealworm larvae (4, 5, 24, 38). The proximate results are also in line with the one reported by Caparros Megido et al. (11) on mealworm larvae cooked with household cooking technique.

Dry matter (DM) content is strictly related with the cooking technique and the corresponding water loss. Unprocessed larvae were not modified in their structure and maintained humidity content naturally present into their body; indeed, uncooked larvae revealed the lowest DM contents. Boiling (under vacuum) and steaming preserved the samples from water evaporation and created an environment where water was kept or even added into the larvae, as highlighted by the low or negative values of cooking losses. Moreover, heat treatment could also lead to protein denaturation that may cause higher water losses due to degeneration of protein structures and losses of capillary forces (12). Apparently, oven cooking at 70°C for 30 min did not deeply affect the mealworm structure and then DM was comparable to the one of UC larvae or even to steamed ones (ST). Higher oven temperature–time combination or microwave cooking induced modifications into the larvae structure that caused water losses and increased DM contents. Frying larvae, both in pan or deeply, induced highest water losses and evaporation increased DM contents, also partially affected by the incorporation of lipids from the frying oil [as reported also in other products, (12)]. Indeed, PF and DF larvae showed the highest content in lipids doubling the UC ether extract content. A similar trend was reported by Caparros Megido et al. (11) in mealworms pan fried in olive oil.

Regarding the crude protein contents, the cooking technique deeply modified the nutritional value of the larvae. Oven-cooked larvae for 10 min at 150°C, due to water loss and concentration of the nutrients, revealed the higher protein content followed by DF and MW. Again, as reported before, water replacement with lipids must be taken into consideration in the total nutritional evaluation of DF larvae.

### *In vitro* Protein Digestibility

Cooking conditions may reduce or increase protein digestibility also in relation to the type of organic material (37). *In vitro* determination highlighted a decreasing percentage of protein digestibility after simulated gastric digestion in all the cooked samples in comparison with unprocessed larvae (UC). Cooking may contribute to the formation of complex organic compounds that are difficult to be digested by pepsin. Indeed, enzymatic proteolysis may be affected by modification that occurred during cooking such as formation of amide bonds or disulfide/dityrosine bridges (39). Between the cooking techniques employed, DF showed the highest reduction in simulated protein gastric digestibility followed by OC150-10, PF, and BO with minor differences to OC70-30, MW, and ST.

*In vitro* protein digestibility increased in all the cooked samples after simulated duodenal digestion, with significant changes in OC70-30, PF, DF, and ST larvae. Uncooked larvae maintained the same percentage during the two *in vitro* steps showing that after gastric digestion, the matrix was not furthermore digested. Increases in cooked larvae digestibility and absence of variation in UC affected the trend in protein digestibility after *in vitro* duodenal digestion with the highest value in OC70-30 followed by UC and ST samples.

Our results were partially in accordance with the one shown by Caparros Megido et al. (11) with some differences related to the methods and cooking techniques employed. Fried larvae decreased protein digestibility mostly in relation to their potential oxidation that occurred during the cooking step. Oxidation products of lipids may create complexes with proteins and modify the chemical structure and functionality of proteins and reduce their enzymatic susceptibility (40).

Protein digestibility is important not only in terms of nutritional value but also in relation to potential harmful risk, mainly associated with the risk of colorectal cancer, as a higher quantity of undigested protein entering the colon could increase potential deleterious effects on colonic epithelial cells (41).

### FA Profile

For all cooking methods, a general decrease in SFA and MUFA content was observed. This observation may be explained by the fact that both FA classes are largely represented in neutral lipids and are more prone to migration (42). However, MUFA showed less variations than SFA; the main differences were due to the oleic acid (C18:1) proportion, which was the most representative FA in UC and a similar concentration was found in ST, OC150-10, and PF. Regarding the frying method, Juárez et al. (12) found in buffalo meat the lowest SFA content due to the incorporation of MUFA (C18:1) from oil; conversely, in the present paper, a similar trend was not found regarding MUFA, whereas an increase of PUFA was recorded in the DF group. Furthermore, the PUFA content was also higher in the ST group, followed by BO, OC150-10, and PF with respect to UC and MW. The ST method better preserved the cellular integrity of larvae and then the membrane FA content. Indeed, the PUFA decrease has been related to triglycerides' unsaturated FA drip losses as reported for the grilled meat (43). Among n3 and n6 FA, great differences were recorded; however, it is not possible to draw a unique trend for each FA. The OC70-30 showed the highest values for both LCP n3 and n6, showing a n6/n3 ratio that is 50- (DF) to 11-fold (UC) lower with respect to the other cooking techniques. Such trend may be modulated by an oxidative process that occurred on unsaturated FA during cooking; accordingly, the IP reached the highest value in OC70-30. Indeed, unsaturated FA are strongly susceptible to oxidation, due to the presence of double bonds. Some authors (44) consider that changes in FA composition that occur during cooking may be overlooked when only total lipid extracts are analyzed. In fact, the thermal hydrolysis, the migration of FA from tissue to other locations, the loss of volatile FA, and the deactivation of enzymes occurred during heating may be responsible for many of the observed changes.

### Oxidative Status

The oxidative status of larvae was widely affected by the cooking processes, with strong differences related to the methods used. In the present research, carbonyls increased with oven cooking (OC150-10 and OC70-30), whereas the values were lower with frying and similar to ST and UC. Such trend was probably due to the time/temperature ratio of cooking because frying consisted of a high temperature (350–370°C) for a few seconds (120 s), and then the effect was comparable to the less impactful methods (i.e., ST). Protein oxidation is the covalent modification of a protein induced either directly by reactive oxygen species or indirectly by reaction with secondary products of oxidative stress. Different groups of amino acids are sensitive to oxidation (45): basic amino acids are oxidized into carbonyls, whereas thiol groups of cysteine can be oxidized with the formation of disulfide bridges (46). In agreement with our findings, Gatellier et al. (47) reported that beef meat heated at 207°C for 300 s showed higher carbonylation with respect to heating the same material for a short burst (60 s) at high temperature [(270°C; (48)]. Similarly, cooking larvae *via* microwave showed a negative effect on protein oxidative status.

Concerning the lipid oxidative status, higher values were recorded in DF and OC150-10. It should be noted that a higher lipid and protein oxidation corresponds to a lower antioxidant concentration (tocols). The best oxidative status was recorded in the ST group, although the tocols content was very low (2.55 μg/g DM), probably due to an involvement of such molecules to counteract the oxidative thrust (49). During cooking, antioxidant defense systems are impaired, and contemporary free radicals are produced, leading to protein, and lipid oxidation (50). In agreement, some cooking processes showed a strong antioxidant/pro-oxidant imbalance that negatively affected the oxidative status of the larvae, particularly both oven methods and microwave cooking. A body of literature reported that microwave might affect mainly protein oxidation, whereas frying (both pan and deep) is involved in lipid oxidation (51). Contrary to conventional heating processes, such as oven cooking or pan frying, electromagnetic microwaves have frequencies between 0.3 and 300 GHz and wavelengths between 1 nm and 1 mm that can penetrate food and directly excite specific molecules by ionic conduction and dipole rotation. The overall effects of these movements lead to an increased kinetic energy of the molecules, resulting in increased temperature and cooking rate. Given that this mechanism allows for intimate interaction with the molecules of the food, generation of reactive oxygen species could result due to disruption of cellular compartmentalization.

Contrary to the trend in protein oxidation, deep frying and cooking at 150°C in an oven for 10 min contributed to a greater increase in lipid oxidation (TBARS) compared to microwave cooking. Some studies have, however, reported contrary results with higher lipid oxidation in microwave compared to frying (52). This discrepancy may be due to the nature of the food involved, as well as the length and temperature of the cooking method. In addition, total fat content and FA profile surely played an important role in lipid oxidation (TBARS).

## Conclusions

*Tenebrio molitor* larvae represent a valid source of nutritional values. Cooking technique could affect (*in vitro*) the protein digestibility, FA profile, and oxidative status of the product. Between the tested cooking techniques, steaming was the less invasive method in terms of nutritional value modifications, lipid–protein oxidations, and *in vitro* protein digestibility. Microwave and oven cooking methods showed mixed effects leading to miscellaneous outcomes. Frying, both in pan and deep frying, induced great modifications in larvae FA profile, with a subsequent decrease in lipid oxidative status. Boiling could also be reported as a mild cooking method despite the fact that some modifications in chemical composition were shown (mainly PUFA and vitamins). Nutritional composition of cooked larvae was also affected by cooking losses that occurred in relation to the method employed, and attention must be paid to maintain certain types of molecules also in relation to the fixed goals. Noteworthy, despite differences highlighted by the cooking techniques, nutritional values of mealworm could meet humans' requirements and increase vitamins and PUFA intakes. The cooking method must be carefully chosen to maintain a high protein digestibility.

## Data Availability Statement

The raw data supporting the conclusions of this article will be made available by the authors, without undue reservation.

## Author Contributions

SMan and FF contributed to conception and design of the study. SMan, SMat, SP, and FF performed the laboratory analysis. SMan and SMat performed the statistical analysis. SMan wrote the first draft of the manuscript. SMat wrote sections of the manuscript. All authors contributed to manuscript revision, read, and approved the submitted version.

## Conflict of Interest

The authors declare that the research was conducted in the absence of any commercial or financial relationships that could be construed as a potential conflict of interest.
